# Tapasin-mediated editing of the MHC I immunopeptidome is epitope specific and dependent on peptide off-rate, abundance, and level of tapasin expression

**DOI:** 10.3389/fimmu.2022.956603

**Published:** 2022-10-31

**Authors:** Denise S. M. Boulanger, Leon R. Douglas, Patrick J. Duriez, Yoyel Kang, Neil Dalchau, Edd James, Tim Elliott

**Affiliations:** ^1^ Centre for Cancer Immunology, Faculty of Medicine, University of Southampton, Southampton, United Kingdom; ^2^ Cancer Research UK (CR-UK) Protein Core Facility, Faculty of Medicine, University of Southampton, Southampton, United Kingdom; ^3^ Microsoft Research, Cambridge, United Kingdom; ^4^ Centre for Immuno-oncology, Nuffield Department of Medicine, University of Oxford, Oxford, United Kingdom

**Keywords:** tapasin, antigen presentation, major histocompatibility class I, peptide repertoire, immunopeptidome, interferon-γ

## Abstract

Tapasin, a component of the major histocompatibility complex (MHC) I peptide loading complex, edits the repertoire of peptides that is presented at the cell surface by MHC I and thereby plays a key role in shaping the hierarchy of CD8+ T-cell responses to tumors and pathogens. We have developed a system that allows us to tune the level of tapasin expression and independently regulate the expression of competing peptides of different off-rates. By quantifying the relative surface expression of peptides presented by MHC I molecules, we show that peptide editing by tapasin can be measured in terms of “tapasin bonus,” which is dependent on both peptide kinetic stability (off-rate) and peptide abundance (peptide supply). Each peptide has therefore an individual tapasin bonus fingerprint. We also show that there is an optimal level of tapasin expression for each peptide in the immunopeptidome, dependent on its off-rate and abundance. This is important, as the level of tapasin expression can vary widely during different stages of the immune response against pathogens or cancer and is often the target for immune escape.

## Introduction

The CD8 T-cell response is an important arm of the immune system against pathogens and cancer, triggered by the recognition of peptide: major histocompatibility complex (MHC) I (pMHC) on the surface of antigen-presenting cells (APC). The abundance of specific pMHC on an APC surface is an important determinant of T-cell function and fate, underpinning the probability of T-cell activation upon T-cell priming, the probability of discharging effector function upon encounter with a target, the maintenance of T-cell memory, and the induction of T-cell exhaustion.

Three factors dominate the relative abundance of pMHC displayed at the cell surface: peptide supply, peptide off-rate from MHC, and peptide editing by tapasin and TAPBPR (1–4).

The amount of peptide being supplied to the ER will be determined by the origin and abundance of the peptide source translation product, how efficiently the peptide is processed by cytosolic enzymes including the constitutive and immuno-proteasome, how efficiently the peptide is transported through TAP, and whether ERAAP is essential or detrimental to the processing of the peptide to its optimum length for loading onto a specific MHC I allele. Peptides from the resulting ER pool compete to load onto MHC I molecules within the peptide loading complex (PLC) made of the TAP transporter (TAP1/TAP2) and two subcomplexes: MHC I-calreticulin and tapasin-ERp57 (5). Tapasin bridges MHC I to TAP and therefore brings the MHC I molecules close to the peptide delivery site (6). It also stabilizes the transporter and maximizes peptide supply to the ER (7), reviewed in (8). Furthermore, tapasin promotes the exchange of low-affinity peptides bound in the MHC I cleft for higher-affinity peptides (9–12), a process known as peptide editing (13).

Peptide off-rate from MHC I is determined by multiple interactions between side chains and backbone residues of the peptide and amino acid residues that line the antigen-binding cleft of the class I molecule in distinctive pockets (denoted A–F) of varying depths and chemistry. Extensive polymorphism among MHC I results in a degree of peptide selectivity based on favorable side chain: pocket interactions. Whereas all MHC I alleles studied to date are loaded more efficiently with their peptide cargo in the presence of tapasin, there is large variability between allelic variants regarding the extent to which this is so (14–18). Consequently, for some alleles (like HLA-B*44:02), tapasin appears to be essential for expression, whereas others (such as B*44:05) have an intrinsic capacity for peptide editing (10–12, 19, 20) and are expressed efficiently in cells that lack tapasin, albeit with a suboptimally edited peptide cargo. These phenomena are captured by a metric “tapasin index,” which is allele specific and refers to the level of pMHC presentation in the presence of tapasin divided by the level of presentation in its absence (18). Alleles that are expressed well in the absence of tapasin (by virtue of their high intrinsic editing capacity) have a low tapasin index, whereas the so-called tapasin-dependent alleles have a high tapasin index.

Tapasin expression levels can vary over orders of magnitude: it can be upregulated following infection and ligation of TLRs or under the influence of inflammatory cytokines such as interferon gamma (IFNγ), IFNα, tumor necrosis factor alpha (TNFα), and interleukin (IL)-4 (21). Similarly, tapasin can be overexpressed in the inflammatory environment surrounding some cancers (22). Conversely, viruses can target/compromise tapasin resulting in a decreased MHC I surface expression. For example, adenovirus E3-19K blocks tapasin from binding to TAP (23, 24), and molluscum contagiosum MC80 targets tapasin for degradation (25). Similarly, many tumors downregulate or lose tapasin expression (26), which correlates with poorer prognosis (27–30), tumor progression and metastasis (31), and lower CD8+ TILs (29, 32). However, in contrast, in salivary gland carcinomas, tapasin upregulation was significantly associated with shorter overall survival (33).

In addition to changes in the tapasin expression level, other parameters determining the level of peptide presentation can also be subject to change during the course of an immune response to a tumor or virus: (a) source protein levels (e.g., an oncoprotein) may vary over orders of magnitude; (b) levels of expression of antigen processing enzymes and TAP may vary and impact peptide supply; (c) individual anchor amino acids that comprise the agretope within a peptide may mutate to enhance or diminish binding to MHC (peptide off-rate), without necessarily affecting the epitope. We captured these elements in a mechanistic model of antigen presentation that is capable of predicting the quantity of distinct pMHC species presented at the surface of an antigen-presenting cell while considering competition between multiple peptides for binding to MHC, peptide supply, binding strength, and tapasin editing (1, 19, 34). In the model, tapasin enhances peptide unbinding to improve peptide optimization without significantly delaying the transit of MHC I to the cell surface. Differences in peptide optimization across MHC class I alleles could be explained by allele-specific differences in MHC I protein dynamics (1, 19). Taken together, these suggest that, mechanistically, tapasin catalyzes the interconversion of MHC I between an (open) intermediate state and a (closed) native state (reviewed in (2)). This is also consistent with structural studies of MHC I in complex with the tapasin homologue TAPBPR (35, 36).

Here, we used the mathematical model to guide an investigation into the compound effect of varying peptide supply, affinity, and tapasin expression on levels of antigen presentation in a physiological cellular model with all its complexity (complete PLC and presence of other MHC I alleles) compared to more restricted *in vitro* studies. By comparing the level of peptide presentation in wild-type cells, in TpnKO cells where the PLC would be disrupted and in TpnKO cells reconstituted with different levels of tapasin expression, this study aims at measuring the effect of tapasin loss or tapasin modulation (up or down) on the level of filtering of peptides of different affinities and abundance.

## Material and methods

### Cells

Wild-type and TpnKO fibroblasts cell lines were produced from primary ear fibroblasts harvested from C57BL/6 wild-type mice or TpnKO mice, respectively, and immortalized by transfection with pSV3-neo plasmid (ATCC, Cat No. 37150) encoding the SV40 T-Ag (34). These were cultured in Roswell Park Memorial Institute (RPMI) 1640 (Lonza, Verviers, Belgium) supplemented with 10% fetal calf serum (FCS) (Globepharm, Guildford, UK), 2 mM glutamine (Lonza), and 50 µM β-mercaptoethanol.

All cells were confirmed to be mycoplasma negative by PCR.

The authenticity of cells was verified by continuous confirmation by flow cytometry that the cells expressed the correct MHC I alleles at the correct relative level of expression.

### Antibodies

The E10 human Fab reagent specific for ASNENMETM-H2Db was expressed in bacteria from a plasmid kindly given by Bennink and Yewdell (37) as described previously (34).

A chimeric 1C3 monoclonal antibody (human variable domains fused to mouse IgG1 constant domains) generated in mammalian cells was used to detect SSLENFRAYV-H2Db (34).

B22 and Y3 mouse monoclonal antibodies, reacting against peptide-loaded H-2Db and H-2Kb, respectively (38, 39), were produced in-house.

Secondary antibodies were goat anti-human IgG (H+L) secondary antibody [GAH-AF647 (Thermo Fisher Scientific, Cat. No. A-21445, RRID : AB_2535862] to detect E10 and goat F(ab′)2-anti-mouse IgG (H+L) secondary antibody [GAM-AF647 (Thermo Fisher Scientific, Cat. No. A-21237, RRID : AB_2535806) to detect 1C3, B22, and Y3.

Anti-mouse tapasin 5D3 hamster monoclonal antibody (BioLegend, Cat. No. 696702, RRID : AB_2687096) was used in WB, followed by goat anti-Armenian hamster IgG (H+L) secondary antibody conjugated to horseradish peroxidase (HRP) (GAHam-HRP, Thermo Fisher Scientific, Cat. No. PA1-32045, RRID : AB_10985178).

### Plasmids

Influenza peptides restricted to H-2Db (SSLENFRAYV, PA224–233) and variants of ASNENMETM (NP366–374) were expressed from Venus/mCherry-ubiquitin-peptide plasmid constructs generated as described in (34).

To generate tapasin-cerulean plasmids, mouse tapasin sequence was amplified from wild-type fibroblasts cDNA by PCR (using primers GATCTCGAGCTCAAGCTTCGAATTCATGAAGCCTCTGCTCCTGCT and GCTCACCATGGTGGCGATGGATCCCTGTGACTTCTTTGAGTTCCTG to generate pTpn-Cer or GCTCACCATGGTGGCGATGGATCCTTACTGTGACTTCTTTGAGTTCC to generate pTpn-STOP) and inserted into mCerulean3-N1, a gift from Michael Davidson (Addgene plasmid, Cat. No. 54730, RRID : Addgene_54730) (40). Plasmid sequences were checked by sequencing using primers CMVF_pCDNA3 (CAACGGGACTTTCCAAAATG), EGFP C R (GTTCAGGGGGAGGTGTG), and EGFP Nrev (CGTCGCCGTCCAGCTCGACCAG).

A KKXX retention signal was inserted at the C-terminus of cerulean in pTpn-Cer to create pTpn-Cer-KKXX by site-directed mutagenesis (QuickChange Site-Directed Mutagenesis kit from Stratagene) using primer GACGAGCTGTACAAGAAGAAGTCTCAATAAAGCGGCCGCGAC and its reverse complement sequence.

### pMHC presentation assay

Wild-type or TpnKO fibroblasts were seeded at 10^5^ cells per well in a six-well plate. When IFNγ treatment was applied, 1 µg of mouse IFNγ (Peprotech, Rocky Hill, USA) was added per well 4 h after seeding. Cells were transfected the following day with TransIT-LT1 (Mirus, Madison, USA) following the manufacturer’s recommendations using 2.5 µg (single transfections) or 2 × 1.25 μg (co-transfections) of plasmid DNA per well. One day after transfection, cells were stained for 45 min on ice with primary reagents, 1C3 hybrid mAb, E10-purified Fab, B22, or Y3-purified mAbs, to detect surface pMHC complexes. After washing, cells were incubated for 45 min with GAH-AF647 (used after Fab primary) or GAM-AF647 (after mouse antibodies including 1C3). Flow cytometry was performed using a Fortessa X20 cytometer (BD, UK), and the data were analyzed using FACS Diva software (BD).

Inhibition of ERAAP was achieved by adding, on transfection day, L-leucinethiol oxidized dihydrochloride (Sigma) (30 µM final concentration).

### Quantitation of tapasin in sorted cells by Western blotting

A set number of cells (transfected or not), as indicated in the figure legend, was sorted using an Aria sorter (BD) according to mCerulean fluorescence intensity, reporting on the level of expression of tapasin. Corresponding cell pellets were lysed by freeze–thawing in phosphate-buffered saline (PBS) and loaded on a 12.5% sodium dodecyl sulfate–polyacrylamide gel electrophoresis (SDS-PAGE) gel in reducing buffer. Separated proteins were transferred onto a nitrocellulose membrane (PROTAN, Amersham). Tapasin was identified by using the anti-tapasin 5D3 antibody and GAHam-HRP, revealed with the SuperSignal West Femto maximum sensitivity substrate from Pierce, and visualized on a UVP BioSpectrum AC Imaging System.

### Brefeldin A decay assay

Cells were seeded and transfected as above. One day after transfection, Brefeldin A was added at 5 μg/ml to block *de novo* transport of MHC I to the cell surface, and the cells were returned to 37°C for 1, 2, or 4 h to follow the decay of surface pMHC complexes. Cells were harvested at time 0, 1, 2, and 4, washed in FACS buffer, and stained with B22 mAb (conformation sensitive anti-H-2Db mAb) and GAM-AF647 to detect peptide-loaded MHC-I molecules. Samples were analyzed by flow cytometry on a Fortessa X20 flow cytometer, and data were analyzed with the Diva software. Mean fluorescence intensity (MFI) values were background deducted by subtracting the MFI value obtained in the unstained control. Half-lives and off-rate constants were then determined by fitting the curves using an exponential trend line in Excel software (Microsoft, USA).

## Results

### Individual peptides have an optimal tapasin bonus that is dependent on both their affinity and abundance

Howarth et al. (10) previously showed that peptide presentation was enhanced by tapasin according to the half-life of the pMHC complex when the different peptides were supplied at the same rate. This enhancement can be expressed as a peptide-specific tapasin bonus, calculated as the ratio of surface presentation in the presence versus the absence of tapasin.

**Figure 1:**
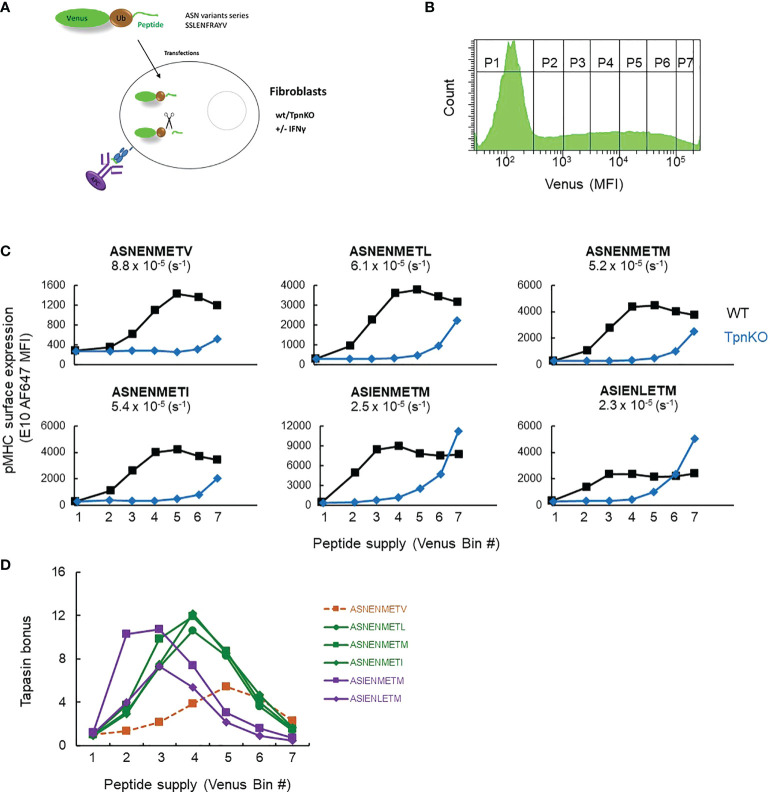
pMHC surface expression at increasing peptide supply and tapasin bonus. **(A)** Experimental setup. Wild-type and TpnKO fibroblasts were transfected with constructs expressing fusion proteins made of a fluorescent protein, ubiquitin, and an ASN variant peptide. pMHC complexes were detected on the cell surface using E10 Fab, recognizing all of the ASN variants bound to MHC I molecules, although with varied affinities ((34), Supplementary Figure S7). **(B)** Cytoplasmic ubiquitin hydrolases cleave the fusion proteins, releasing an equimolar ratio of peptide and fluorescent protein. The increasing level of venus expression in gates P1–P7 corresponds to increasing peptide supply. **(C)** Surface expression of ASN variants, from lower-affinity ASNENMETV to the highest-affinity ASIENLETM peptide (off-rates calculated in (34) are indicated for each peptide), in wild-type (black squares) and TpnKO (blue diamonds) fibroblasts at increasing peptide supply recorded by the venus MFI in the different gates indicated by their bin number. As E10 Fab has a different affinity for each ASN variant complex, the staining intensity varies between variants and cannot be compared between them. The aim of this experiment is to compare staining between wild-type and TpnKO cells for each ASN variant. **(D)** The tapasin bonus, ratio of pMHC presentation in the presence of tapasin on wild-type cells over presentation on TpnKO cells, was plotted at increasing peptide supply.

Peptide editing by tapasin requires iterations of binding and unbinding of pMHC to tapasin, and peptides can enter this cycle by binding to MHC that is either tapasin bound or not, which is a mechanistic feature of the peptide filter model that we published previously (1). A qualitative prediction from this is that, as peptide supply increases, peptide binding to MHC will occur more frequently than tapasin binding to MHC (as long as tapasin levels are limiting), and as a result, the tapasin bonus will diminish. Moreover, the tapasin bonus would vary depending on the tapasin index of the MHC allele, the peptide affinity, and the peptide supply (**Supplementary Figure S1**). We therefore tested this prediction experimentally and varied peptide supply for a number of peptides of known affinity over a range that might be encountered *in vivo* for viral or tumor antigens (we previously estimated the maximum copy number of the fluorescent-Ub-peptide fusion protein, the source for the peptide supply, to reach a maximum of 10^8^ molecules per cell [(34) **Supplementary Figure S1B, E, F**], which is similar to the maximum copy number per cell of some proteins (41, 42)), then measured the tapasin bonus for individual peptides. We measured the surface expression of a number of peptide variants of the Influenza virus peptide ASNENMETM with mutations at MHC I anchor positions affecting their binding affinity to murine MHC I H-2Db (ASN variants): ASNENMETV (off-rate: 8.8 × 10^−5^ s^−1^), ASNENMETL (6.1 × 10^−5^), ASNENMETM (5.2 × 10^-5^), ASNENMETI (5.4 × 10^−5^), ASIENMETM (2.5 × 10^−5^), ASIENLETM (2.3 × 10^−5^) as measured in (34). The peptides were expressed endogenously as fusion proteins comprising a fluorescent protein (venus), ubiquitin, and the peptide sequence (34) (**Figure 1A**). The amount of peptide generated in the cytoplasm (peptide supply), after cleavage of the polyprotein by ubiquitin hydrolase, is directly correlated with the amount of fluorescent protein, which can be measured by flow cytometry (**Figure 1B**). The broad range of venus expression allowed the definition of seven bins of increasing MFI or peptide supply (P1 to P7, **Figure 1B**), allowing the expression of cell surface pMHC abundance to be reported as a function of intracellular peptide abundance in both wild-type and TpnKO fibroblasts for each ASN variant (**Figure 1C**).

**Figure 1D** shows that the tapasin bonus for each peptide variant is dependent not only on peptide affinity but also on peptide supply as predicted (**Supplementary Figure S1**, right panels). Thus, when peptide supply rate is low (bin 2), the tapasin bonus for high-affinity ligands such as ASIENMETM can be 10 times higher than for low-affinity ligands like ASNENMETV. In the context of a viral infection, this means that at the early stages of infection when few viral peptides are available, tapasin will enhance the presentation of high-affinity peptides over abundant self-peptides of lower affinity. As the kinetics of presentation is crucial in determining immunodominance (43), high-affinity epitopes would therefore be more likely to become immunodominant during early infection.

When peptide supply rates are high (bin 5), the tapasin bonus for high-affinity peptides is low, as those peptides are presented efficiently even in the absence of tapasin (**Figure 1C**).

### Individual peptide presentation in the absence of tapasin depends on both affinity and abundance and is enhanced by interferon-γ

All ASN variants were detected on the surface of TpnKO cells when the peptides were abundantly expressed, with an increased presentation as the peptide affinity increased (**Figure 1C**). In some experiments, high expression levels of high-affinity peptides (ASIENMETM and ASIENLETM) achieved the same level of presentation in the absence of tapasin as in its presence (**Figure 1C**). High antigen expression can therefore drive peptide presentation on tapasin-deficient cells.

**Figure 2:**
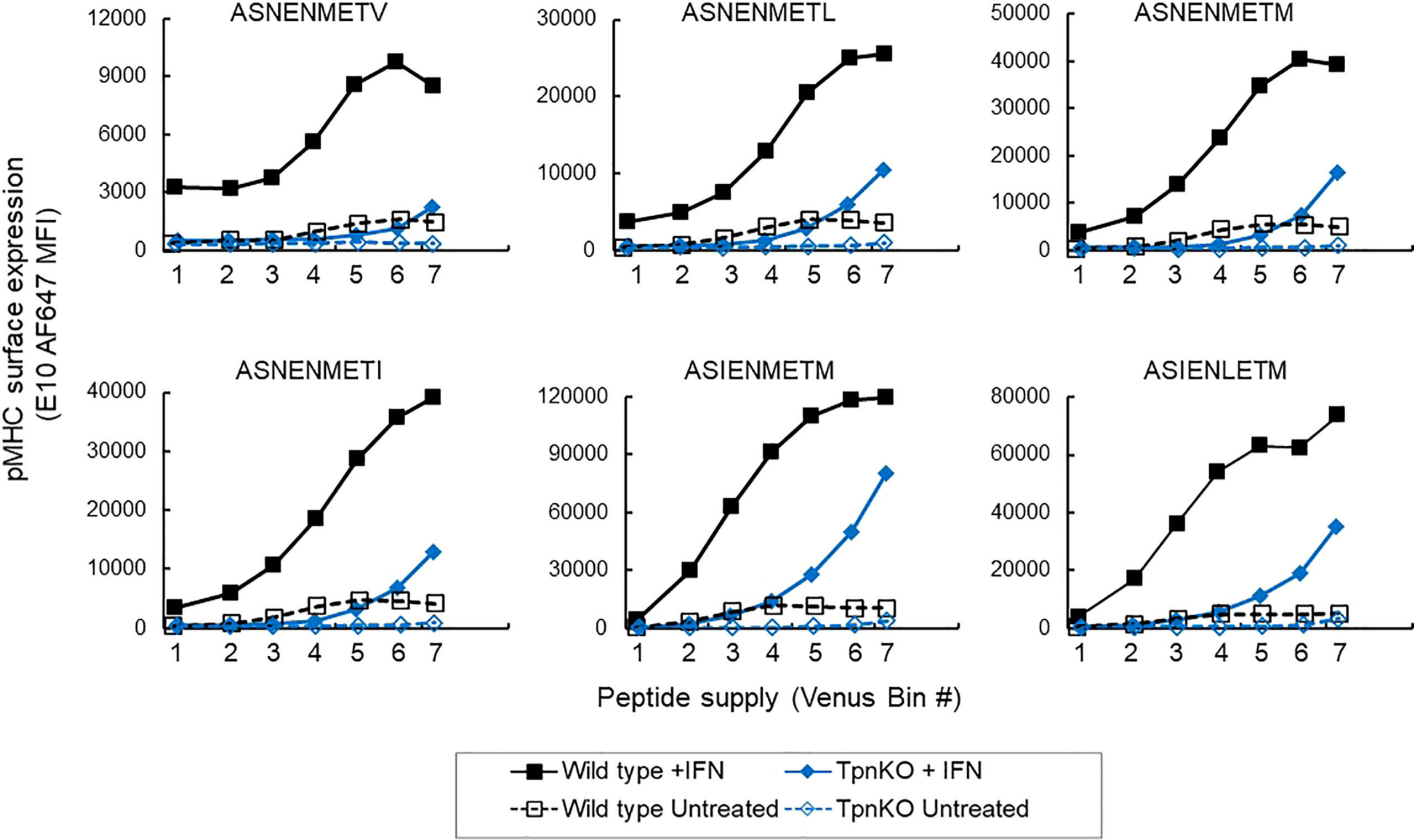
pMHC surface expression at increasing peptide supply on cells treated with IFNγ. Surface presentation of ASN variants (from the lower-affinity ASNENMETV to the highest-affinity ASIENLETM peptide) on wild-type (black squares) and TpnKO (blue diamonds) fibroblasts were plotted at increasing peptide supply on the x-axis (venus MFI in each gate). Surface presentation on cells treated with IFNγ (solid fill) can be compared to presentation in untreated cells (open symbols) showing that the presentation of abundant peptides in TpnKO cells treated with IFNγ can exceed the presentation in untreated wild-type cells. This figure is a repeat of **Figure 1C**, with all peptides tested in a single experiment.

Under inflammatory conditions, in infections or cancer, IFNγ induces an increased expression of MHC I and proteins involved in antigen processing and presentation (APP), including tapasin, resulting in an increase in antigen processing, transport, and presentation. In wild-type fibroblasts stimulated with IFNγ, steady-state MHC I and tapasin RNA levels, measured by real-time quantitative PCR (RT-qPCR), were increased approximately 4 and 8 times, respectively (data not shown, schematic in **Supplementary Figure S2**), whereas TAP1 and TAP2 increased approximately 35 and 16 times. In comparison, in TpnKO fibroblasts, MHC I, TAP1, and TAP2 RNA levels increased by 3, 10, and 7 times. Increased expression of these proteins resulted in an increased surface presentation of all ASN variant peptides, not only in wild-type cells, as shown previously (34), but also by a factor of 10–20 in TpnKO cells, reaching and exceeding the levels observed on untreated wild-type cells (**Figure 2**). Thus, for cancer immunotherapy, strategies for increasing intra-tumoral IFNγ expression could improve antigen presentation, not only in tumors where MHC I and/or tapasin downregulation results from regulatory alterations that can be reversed by IFNγ treatment but, importantly, also in tumors that have permanently lost tapasin expression.

### Individual peptides have an optimal level of tapasin expression

Given that individual peptides, defined by their off-rate and their abundance, have an optimal tapasin bonus, it follows that optimal presentation of a given peptide might be achieved by tuning the level of tapasin expression. This is important because levels of tapasin expression can be either dramatically increased in the presence of inflammatory cytokines (eightfold increase after IFNγ treatment in wild-type fibroblasts) or decreased *via* various mechanisms in virus-infected cells or cancer (where expression of tapasin can be downregulated or completely lost), thereby regulating the presentation of crucial protective epitopes.

**Figure 3:**
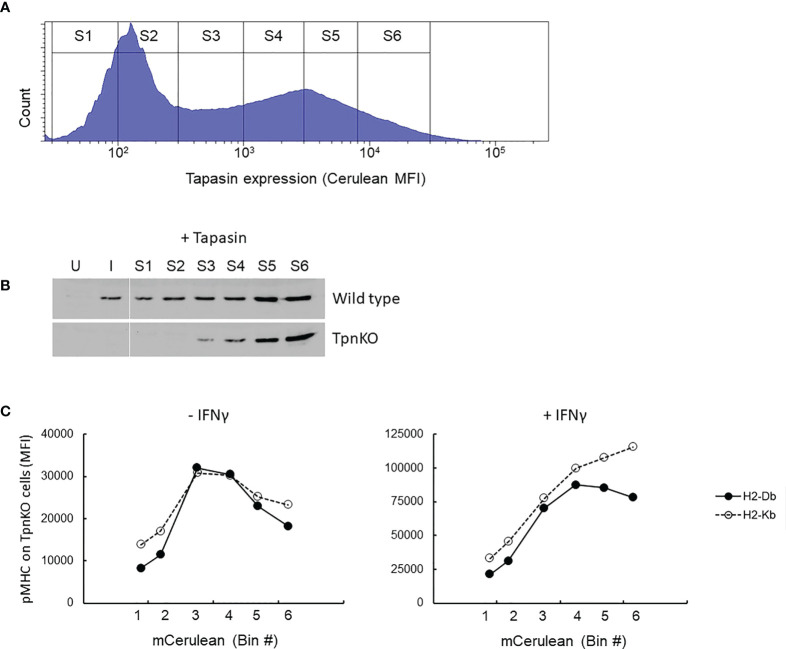
pMHC surface presentation at increasing levels of tapasin expression. **(A)** TpnKO cells were co-transfected with plasmids (pTpn-STOP and mCerulean3-N1 plasmids) expressing tapasin and mCerulean, reporting indirectly the level of tapasin expression, and divided in six gates of increasing expression level (S1–S6). **(B)** Tapasin expression level was measured in lysates from 50,000 wild-type or TpnKO cells transfected with tapasin and sorted in gates S1–S6 (lanes S1–S6), compared to 50,000 untreated untransfected sorted cells (U) and IFNγ treated (I) cells. (In wild type untransfected cells, 70,000 cells would have been necessary to visualize a faint band, but in these conditions, Tpn expression levels above S4 were oversaturated; data not shown). **(C)** H-2Db (filled circles) and H-2Kb (empty circles dotted line) surface levels on TpnKO cells expressing increasing amount of transfected tapasin reported by cerulean MFI on the x-axis (untreated cells on the left; IFNγ treated cells on the right).

This phenomenon can be simulated (**Supplementary Figure S3**), showing that the optimal presentation of high-affinity peptides was achieved in the presence of a higher tapasin expression level compared to lower-affinity peptides and that surface expression of all peptides decreased over a certain level of tapasin expression. To investigate this experimentally, TpnKO cells were transfected with a tapasin-expressing plasmid and a quantifiable fluorescent reporter. As cerulean fluorescence was quenched when expressed as a fusion protein with tapasin (see **Supplementary Figure S4**), we co-transfected cells with tapasin and cerulean expressed from separate plasmids (pTpn-STOP and mCerulean3-N1 plasmids) (**Supplementary Figure S4A**). To check that the tapasin expression spanned physiological levels, we sorted identical numbers of cells from gates of increasing cerulean MFI (**Figure 3A**, sorting gates S1–S6) and semi-quantified the level of tapasin expression by Western blotting (**Figure 3B**). Expression levels ranged from the levels seen in IFNγ-induced wild-type fibroblasts (**Figure 3B**, lane I) to the low levels that can be seen in some human tumor cell lines (**Supplementary Figure S5**). Tapasin was barely detectable in wild-type cells in the absence of IFNγ induction (**Figure 3B**, lane U) but was clearly induced by IFNγ. Therefore, we observed a clear difference in pMHC presentation between wild-type and TpnKO cells (**Figure 1**), even though wild-type cells expressed only a low level of tapasin.

As expected, as transfected tapasin was expressed under the control of the CMV promoter, its expression level was not increased under IFNγ treatment (**Supplementary Figure S4D**).

**Figure 3C** shows total pMHC surface presentation at increasing tapasin expression levels. In the absence of tapasin, surface H-2Db and H-2Kb expression was low and escalated with increasing levels of ectopic tapasin expression to a maximum of roughly fourfold above the baseline before dropping as tapasin expression increased further. After IFNγ treatment, this bell-shaped expression profile was less pronounced for both H-2 alleles, particularly H-2Kb, which did not reach maximum expression even at the highest levels of tapasin expression achieved (**Figure 3C**, right panel). We speculate therefore that the decline in surface expression at high levels of tapasin is at least partially due to limiting MHC supply, which seems to be restored by IFNγ more efficiently for H2-Kb than for H2-Db (**Figure 3C**, right panel).

### Elevated tapasin expression results in more intense peptide filtering

The mechanism underpinning the observation that tapasin titration curves were concave and not plateaued (**Figure 3C**; also seen in wild-type cells, **Supplementary Figure S6**) was investigated further. According to our model, increased tapasin expression should lead to a greater flux through the MHC-tapasin-bound loading pathway, and *in extremis*, this should lead to the release of a less-diverse immunopeptidome with a higher average affinity to the cell surface (19). That is to say, the peptide repertoire should experience more intense filtering.

We tested this idea in two ways: first, we measured the average peptide cargo stability of H-2Db in cells expressing increasing levels of tapasin, and second, we quantified the loading and presentation of peptides with different affinities as a function of tapasin expression level.

**Figure 4:**
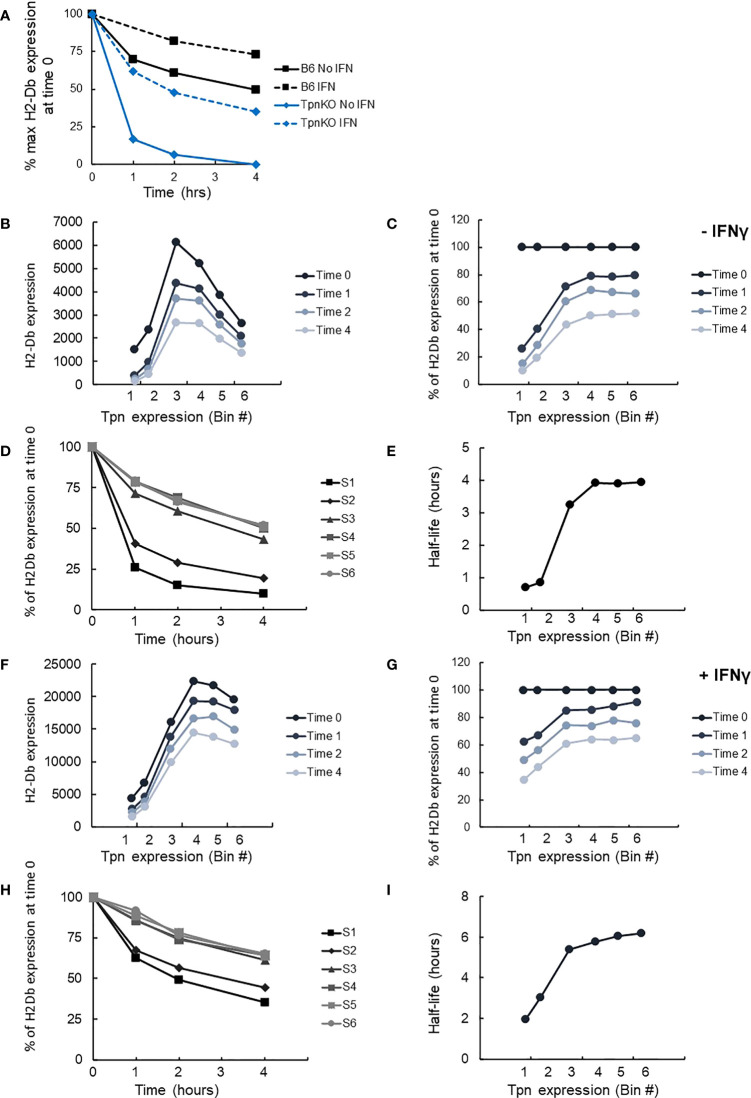
pMHC stability at increasing tapasin expression levels assessed by BFA decay assay. **(A)** The level of H-2Db pMHC surface expression was followed over time by staining with the B22 mAb and plotted as a percentage of the level measured at time 0 on wild-type cells (black squares) or TpnKO cells (blue diamonds) that were either untreated (plain line) or IFNγ treated (dotted lines). **(B)** pMHC (H-2Db) expressed on transfected TpnKO cells after 0, 1, 2, or 4 h decay was plotted at increasing levels of tapasin expression. **(C)** Plotting pMHC expression as a percentage of the level of expression at time 0 showed increased stability of the peptide cargo as tapasin expression increases. **(D)** pMHC stability was plotted over time for each tapasin expression levels in gates S1–S6. Half-lives were calculated from these curves using Excel trendline exponential decay and plotted in Panel **(E)** at increasing tapasin expression levels on the x-axis. Panels **(F–I)** shows the corresponding plots for IFNγ-treated cells.

As demonstrated previously, pMHCs presented on the surface of wild-type fibroblasts were more stable than those presented on TpnKO, as shown by following the decay of pMHC surface levels over time on cells treated with brefeldin A, which blocks transport to the cell surface of newly loaded MHC I molecules (**Figure 4A**). Thus, tapasin increases pMHC half-life from 35 min to 4 h in the absence and from 2 h to well over 4 h in the presence of IFNγ.

We next compared H-2Db stability as a function of tapasin expression level, using the same brefeldin A-decay assay. H2-Db surface expression followed the same bell-shape pattern as shown previously (**Figure 3C**) at time 0 and after 1, 2, or 4 h of decay (**Figure 4B**). Plotting the surface expression as a percentage of expression at time 0 clearly demonstrated that the peptide cargo increased in stability as tapasin expression increased until reaching a plateau by bin 4 (**Figure 4C**). At this level of tapasin expression, the half-life of the peptide cargo presented on transfected TpnKO cells was about 4 h, similar to the half-life of the peptide repertoire presented by wild-type cells (**Figure 4D, E**). A similar response was observed for IFNγ-stimulated cells (**Figures 4F–I**), albeit with different start and endpoints as noted above.

We next measured peptide filtering as a function of tapasin expression directly by quantifying the selection and presentation of peptides with different kinetic stabilities (off-rates) with increasing tapasin expression. We measured the relative surface expression of three peptides of increasing affinity, namely, ASNENMETV, ASNENMETM, and ASIENMETM, expressed individually as venus fusion proteins allowing quantification of their intracellular supply. In addition to measuring the level of presentation of each peptide using pMHC-specific reagents, we also measured total cell surface H2-Db expression.

**Figure 5:**
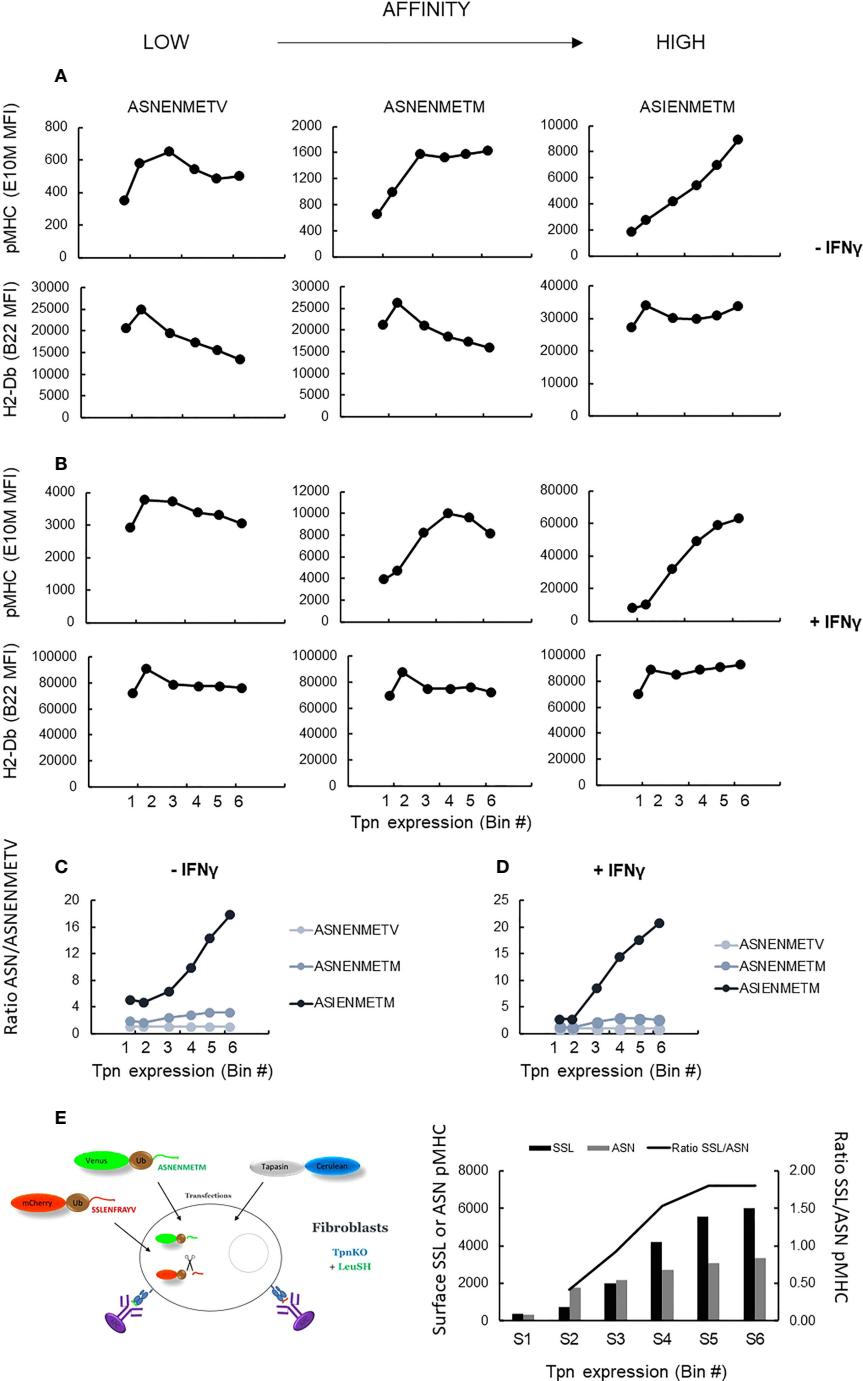
pMHC surface presentation of ASN variants at increasing tapasin expression levels. **(A)** TpnKO cells were transfected with tapasin (and mCerulean) and venus fusion proteins expressing either ASNENMETV, ASNENMETM, or ASIENMETM peptides. ASN pMHC (top panels) and total pMHC (H-2Db) (bottom panels) were measured at increasing tapasin expression levels on the x-axis. **(B)** Same in IFNγ-treated cells. **(C, D)** Plotting the ratio of presentation of ASNENMETM (medium affinity) and ASIENMETM (high affinity) compared to the presentation of ASNENMETV (lowest-affinity peptide) revealed the increased filtering advantage towards the high-affinity peptide as tapasin expression increases in untreated cells **(C)** and IFNγ-treated cells **(D)**. **(E)** TpnKO cells were transfected with venus-Ub-ASNENMETM and mCherry-Ub-SSLENFRAYV and with tapasin-STOP and cerulean. Surface presentation of each peptide was determined by separate staining and recorded for increasing tapasin expression in the six gates shown in **Figure 3A**. Surface presentation of SSLENFRAYV (black histograms) increased in gates S1–S6 with increasing levels of tapasin expression. Surface presentation of ASNENMETM (gray) increased to a lower rate. The increasing ratio of SSL/ASN presentation is shown by the black line.

**Figure 5** shows the level of presentation of each peptide, generated from the intracellular fusion protein, with increasing levels of tapasin expression (top row) compared to total H-2Db expression (bottom row), in the presence (**Figure 5B**) or absence (**Figure 5A**) of IFNγ stimulation. These data were expressed as a ratio of the presentation of each peptide relative to the presentation of the lower-affinity peptide (**Figures 5C, D**). These plots clearly show that the selection of the high-affinity peptide ASIENMETM over the intermediate- and low-affinity peptides is greater with increasing tapasin expression in both the absence and presence of IFNγ (where H-2Db expression is four to five times higher). Thus, the relative presentation of ASNENMETV : ASNENMETM : ASIENMETM is approximately 1:2:10 when tapasin expression is low (MFI, 10^2^) and rises to 1:6:40 when tapasin expression is high (MFI, 10^4^).

To demonstrate directly that this was a result of peptide editing, we measured tapasin-dependent peptide filtering using simultaneous expression of two intracellular competing peptides as described previously (34). Cells expressing both peptides were gated and analyzed, at increasing tapasin expression levels in the six gates used previously (**Figure 3B**), for their level of surface expression for both peptides (**Figure 5E**, histograms). The ratio of SSLENFRAYV/ASNENMETM surface presentation (**Figure 5E**) shows the extent to which high-affinity peptide SSLENFRAYV is selected in preference to intermediate-affinity ASNENMETM in the presence of increasing levels of tapasin. Thus, over the range of tapasin levels investigated, the ratio of high-/intermediate-affinity peptide occupying surface pMHC went from approximately 0.5 to nearly 2 (**Figure 5C**, y-axis on the right).

Taken together, these experiments demonstrate that increasing tapasin expression correlates with enhanced peptide filtering, leading to a higher proportional representation of high-affinity peptides at the expense of lower-affinity peptides.

## Discussion

Tapasin enhances the presentation of high-affinity peptides on the cell surface, providing stable pMHC complexes ideal for inducing an efficient cytotoxic T-cell response. It is therefore not surprising that tapasin is targeted by viruses as a means of escaping immune detection and is frequently downregulated in tumors that have escaped control by CTLs. Tumors often have a complex pattern of altered expression of APP proteins, making it difficult to differentiate the effect of individual components on the downregulation of MHC-I-restricted antigen presentation. For instance, Thuring et al. (28) showed that a whole cluster of APP was downregulated in 11 glioma cell lines (MHC I heavy chain, β2m, tapasin, TAP1, TAP2, LMP2, and LMP7), although the protein that most strongly correlated to HLA-I levels was tapasin. By isolating tapasin function in our experimental system, we have been able to investigate directly and quantitatively the impact of tapasin expression levels on peptide editing and presentation.

We showed that tapasin editing of the peptide repertoire is a dynamic process, modulated by peptide affinity and supply rate and by the level of tapasin expression. When tapasin is limiting, peptide filtering is low, allowing the presentation of lower-affinity peptides. These are edited out of the repertoire as tapasin level increases and peptide supply becomes limiting: a situation where filtering is high and leads to a more refined repertoire. We also observed that at high tapasin levels, fewer loaded MHC I get to the surface. Mechanistically, this can be explained as a gradual increase in the probability of a pMHC encountering a tapasin molecule compared to egression from the ER. Tapasin-associated pMHC is both prevented from egression and subjected to further iterations of peptide editing. This phenomenon can be simulated with the peptide-filter relation model (1, 19) and illustrates the importance of the tapasin bind–release cycle in editing and cell surface presentation. This is consistent with a catalytic cycle for peptide editing in which MHC I molecules exist in two states, namely, “open” and “closed” and cycles between tapasin bound and tapasin unbound—the state that samples the stability of bound peptide cargo being “closed” (19) and binds to tapasin poorly (2, 11, 44). MHC I loaded with relatively stable peptides will dissociate from tapasin and have a greater probability of egressing to the cell surface, whereas peptides that do not stabilize the closed conformation of MHC I will allow it to open and release the suboptimal peptide. Tapasin functions by catalyzing interconversion between the two states, presumably by stabilizing a structural intermediate (perhaps similar to the one observed for TAPBPR-bound pMHC), thereby increasing the flux through the peptide-bound closed state. This will in turn accelerate the rate of peptide exchange. Increasing the flux through this tapasin-bound state, by simply tuning up the level of tapasin expression, will intensify peptide filtering, resulting in fewer peptides, but of higher affinity, being presented at the cell surface.

Interestingly, the fact that editing is sensitive to the number of tapasin molecules expressed in the early secretory pathway implies that either MHC I can load in “sub-stoichiometric” PLC and/or that tapasin can have an editing and ER retention function outside the PLC.

Saturating the tapasin pathway by increasing peptide supply will lead to relatively more loading *via* a tapasin-independent pathway where the degree of peptide editing depends on the intrinsic properties of the particular MHC I allele. This will consequently lower the tapasin bonus as shown in **Figure 1**—and we would predict that the degree to which this occurs will depend on the relative “tapasin dependency” of the MHC I allele in question: a large reduction for tapasin-dependent alleles, a small reduction for tapasin-independent alleles. We, and others, have previously shown that the tapasin index is MHC I allele dependent (15–18, 45). Thus, some alleles derive low benefit with regard to peptide editing from tapasin, whereas others are dependent on tapasin for their peptide loading and cell surface expression. The H-2Db molecule studied here is moderately dependent (46, 47) and therefore well suited for investigations into the impact of tapasin expression on antigen presentation.

Here, we show that for a single MHC I allele, at the level of individual peptide presentation, tapasin dependence varies according to the peptide supply and off-rate and that there is a different optimal level of tapasin expression, or tapasin bonus, for each peptide depending on its k_off_ and supply rate. This also means that the repertoire of presented peptides (immunopeptidome) is likely to change as tapasin expression is tuned. This could be a significant factor at the level of peptide presentation on tumors expressing different levels of tapasin. In addition, given the differing levels of tapasin dependence among HLA alleles, it is likely that the immunopeptidomes presented by MHC I with a low tapasin index might be less susceptible to fluctuations in tapasin expression.

In the absence of tapasin, competition from high-affinity peptides is lower and allows lower-affinity peptides supplied in high amounts to be presented more efficiently. Immunodominant or protective epitopes can therefore appear or disappear depending on their intrinsic characteristics (48) and on the level of tapasin. For example, when tapasin is downregulated, the presentation of survivin and CEPP55-derived epitopes is reduced in the SW480 colon cancer cell line, and the survivin 2B epitope is lost in the LHK2 lung cancer cell line (29). Conversely, the presentation of an MUC1 epitope was reduced after transfection of tapasin in the Pan02-MUC1 tumor cell line (49).

The overexpression of tapasin can also lead to an overall reduced peptide presentation affecting primarily peptides of low or medium affinity. The presentation of protective epitopes of lower affinity can therefore be reduced when tapasin expression is enhanced. The overexpression of tapasin could even constitute another immune escape mechanism in tumors, and interestingly, elevated tapasin expression has been observed in tumor tissues and cell lines including acinic cell carcinomas (33), multiple myeloma (50), and prostate cancer (51). Conversely, Romero et al. (52) described a subclone of the GR9 mouse fibrosarcoma cell line that had increased transcription of tapasin, H-2D, β2m, and TAP2 but showed decreased surface expression of H-2D, suggesting a scenario of tapasin overexpression similar to the one that we contrived (**Figure 3**). When injected into mice, tumors resulting from that clone grew more slowly than clones where tapasin expression was lower yet had a higher surface expression of H-2 and suggests that this tumor is made more antigenic by enhanced peptide filtering. We have also identified one lung cancer cell line (SK-LU-1) expressing high levels of tapasin (**Supplementary Figure S5**).

We showed at the level of individual peptide presentation that tapasin enhanced the presentation of low copy numbers of high-affinity peptides, but when these were supplied in abundance, tapasin editing was less important for their presentation, and the tapasin bonus declined. As the immune system has evolved mostly under the pressure of pathogens, this means that the presentation of a minimal amount of high-affinity peptides produced early on during an acute infection is optimized due to the presence of tapasin. This would give an advantage to high-affinity peptides to become immunodominant, as the kinetic of epitope presentation is crucial to establish immunodominance (43). However, in chronic viral infections and in cancer, lower-affinity but more abundant peptides could start to dominate on the cell surface.

We also showed (**Figure 1**) that pMHC presentation in the absence of tapasin is governed by the abundance and off-rate of the peptides and that high antigen expression can lead to epitope presentation even on tapasin-deficient cells and even if the peptide has a lower affinity. Targeting abundant peptides, including lower-affinity peptides, rather than considering only rare high-affinity epitopes might therefore be a valid strategy for developing vaccines that could treat tumors that have or are likely to eventually downregulate tapasin expression.

MHC I surface expression can be downregulated by two major mechanisms: genetic defects in MHC I or β2m sequences resulting in irreversible loss of MHC I expression in “hard lesions” or MHC I downregulation resulting from regulatory alterations of MHC I or APP expression in “soft lesions.” Antigen presentation can often be reversed by IFNγ in the latter, but our data suggest that IFNγ could also enhance MHC I surface expression in cells permanently lacking tapasin (**Figure 1**). IFNγ treatment increased the stability of the peptide cargo even in the absence of tapasin (**Figure 4A**), suggesting other contributing factors. Given our finding that in the absence of tapasin, the unedited peptide repertoire is likely to be dominated by peptide affinity and abundance, this result suggests an increased supply of higher-affinity peptides in the presence of IFNγ. Such a situation might arise from both qualitative (peptide generation dominated by immunoproteasome rather than constitutive proteasome) and quantitative (enhanced peptide transport) factors supported by an increased expression of proteasome subunits and of the TAP1 and 2 proteins. In addition, increased expression of TAPBPR could also contribute to the editing of the peptide repertoire resulting in increased stability of the peptide cargo. Moreover, in cells expressing tapasin, IFNγ treatment would not only enhance pMHC presentation but could also broaden the response, as our results suggest that in the presence of IFNγ, tapasin improves the presentation of both high- and medium-affinity peptides, as long as these are processed equally well with or without IFNγ (53).

Altogether, our data suggest that, depending on the affinity and abundance of a peptide, there is a sweet spot of optimal tapasin expression that could be modulated for optimal peptide presentation and exploited in immunotherapy.

## Data availability statement

The original contributions presented in the study are included in the article/**Supplementary Material**. Further inquiries can be directed to the corresponding authors.

## Ethics statement

The studies involving animals were reviewed and approved by the University of Southampton Faculty of Medicine Ethics committee (ERGO No 65132).

## Author contributions

The study was designed by TE and DB. ND advised on the design based on modeling and conducted modeling work. DB and YK conducted experimental work and analysis. TE and EJ advised on experiments. LD and PD optimized reagents. DB, ND, EJ, and TE wrote the manuscript. All authors contributed to the article and approved the submitted version.

## Funding

This work was supported by Cancer Research UK Programme Grant A16997 awarded to TE and EJ.

## Acknowledgments

We would like to thank J. Neefjes and J. Bennink for kindly providing us with plasmid reagents. We are grateful to Nasia Kontouli for her expert assistance in producing the chimeric antibody reagents and to Richard Jewell and Carolann McGuire from the flow cytometry facility for their help with the Aria. We also thank Dr. Andy Van Hateren for the critical reading of the manuscript.

## Conflict of interest

The authors declare that the research was conducted in the absence of any commercial or financial relationships that could be construed as a potential conflict of interest.

## Publisher’s note

All claims expressed in this article are solely those of the authors and do not necessarily represent those of their affiliated organizations, or those of the publisher, the editors and the reviewers. Any product that may be evaluated in this article, or claim that may be made by its manufacturer, is not guaranteed or endorsed by the publisher.

## References

[1] Dalchau N Phillips A Goldstein LD Howarth M Cardelli L Emmott S. A peptide filtering relation quantifies MHC class I peptide optimization. PloS Comput Biol (2011) 7(10):e1002144. doi: 10.1371/journal.pcbi.1002144 22022238 PMC3195949

[2] van Hateren A Elliott T. The role of MHC I protein dynamics in tapasin and TAPBPR-assisted immunopeptidome editing. Curr Opin Immunol (2021) 70:138–43. doi: 10.1016/j.coi.2021.06.016 34265495

[3] Hermann C van Hateren A Trautwein N Neerincx A Duriez PJ Stevanovic S. TAPBPR alters MHC class I peptide presentation by functioning as a peptide exchange catalyst. Elife (2015) 4:1–22. doi: 10.7554/eLife.09617 PMC4718805 26439010

[4] Abelin JG Keskin DB Sarkizova S Hartigan CR Zhang W Sidney J. Mass spectrometry profiling of HLA-associated peptidomes in mono-allelic cells enables more accurate epitope prediction. Immunity (2017) 46(2):315–26. doi: 10.1016/j.immuni.2017.02.007 PMC5405381 28228285

[5] Blees A Januliene D Hofmann T Koller N Schmidt C Trowitzsch S. Structure of the human MHC-I peptide-loading complex. Nature (2017) 551(7681):525–8. doi: 10.1038/nature24627 29107940

[6] Sadasivan B Lehner PJ Ortmann B Spies T Cresswell P. Roles for calreticulin and a novel glycoprotein, tapasin, in the interaction of MHC class I molecules with TAP. Immunity (1996) 5(2):103–14. doi: 10.1016/S1074-7613(00)80487-2 8769474

[7] Lehner PJ Surman MJ Cresswell P. Soluble tapasin restores MHC class I expression and function in the tapasin-negative cell line .220. Immunity (1998) 8(2):221–31. doi: 10.1016/S1074-7613(00)80474-4 9492003

[8] Van Hateren A James E Bailey A Phillips A Dalchau N Elliott T. The cell biology of major histocompatibility complex class I assembly: Towards a molecular understanding. Tissue Antigens (2010) 76(4):259–75. doi: 10.1111/j.1399-0039.2010.01550.x 21050182

[9] Williams A Peh CA Elliott T. The cell biology of MHC class I antigen presentation. Tissue Antigens (2002) 59(1):3–17. doi: 10.1034/j.1399-0039.2002.590103.x 11972873

[10] Howarth M Williams A Tolstrup AB Elliott T. Tapasin enhances MHC class I peptide presentation according to peptide half-life. Proc Natl Acad Sci U.S.A. (2004) 101(32):11737–42. doi: 10.1073/pnas.0306294101 PMC511045 15286279

[11] Chen M Bouvier M. Analysis of interactions in a tapasin/class I complex provides a mechanism for peptide selection. EMBO J (2007) 26(6):1681–90. doi: 10.1038/sj.emboj.7601624 PMC1829385 17332746

[12] Wearsch PA Cresswell P. Selective loading of high-affinity peptides onto major histocompatibility complex class I molecules by the tapasin-ERp57 heterodimer. Nat Immunol (2007) 8(8):873–81. doi: 10.1038/ni1485 17603487

[13] Thomas C Tampe R. MHC I chaperone complexes shaping immunity. Curr Opin Immunol (2019) 58:9–15. doi: 10.1016/j.coi.2019.01.001 30771631

[14] Greenwood R Shimizu Y Sekhon GS DeMars R. Novel allele-specific, post-translational reduction in HLA class I surface expression in a mutant human b cell line. J Immunol (1994) 153(12):5525–36. 7989754

[15] Peh CA Burrows SR Barnden M Khanna R Cresswell P Moss DJ. HLA-B27-restricted antigen presentation in the absence of tapasin reveals polymorphism in mechanisms of HLA class I peptide loading. Immunity (1998) 8(5):531–42. doi: 10.1016/S1074-7613(00)80558-0 9620674

[16] Lan H Abualrous ET Sticht J Fernandez LMA Werk T Weise C. Exchange catalysis by tapasin exploits conserved and allele-specific features of MHC-I molecules. Nat Commun (2021) 12(1):4236. doi: 10.1038/s41467-021-24401-4 34244493 PMC8271027

[17] Rizvi SM Salam N Geng J Qi Y Bream JH Duggal P. Distinct assembly profiles of HLA-b molecules. J Immunol (2014) 192(11):4967–76. doi: 10.4049/jimmunol.1301670 PMC4117407 24790147

[18] Bashirova AA Viard M Naranbhai V Grifoni A Garcia-Beltran W Akdag M. HLA tapasin independence: Broader peptide repertoire and HIV control. Proc Natl Acad Sci U.S.A. (2020) 117(45):28232–8. doi: 10.1073/pnas.2013554117 PMC7668082 33097667

[19] Bailey A Dalchau N Carter R Emmott S Phillips A Werner JM. Selector function of MHC I molecules is determined by protein plasticity. Sci Rep (2015) 5:14928. doi: 10.1038/srep14928 26482009 PMC5224517

[20] van Hateren A Bailey A Werner JM Elliott T. Plasticity of empty major histocompatibility complex class I molecules determines peptide-selector function. Mol Immunol (2015) 68(2 Pt A):98–101. doi: 10.1016/j.molimm.2015.03.010 25818313 PMC4726658

[21] Abarca-Heidemann K Friederichs S Klamp T Boehm U Guethlein LA Ortmann B. Regulation of the expression of mouse TAP-associated glycoprotein (tapasin) by cytokines. Immunol Lett (2002) 83(3):197–207. doi: 10.1016/S0165-2478(02)00104-9 12095710

[22] Seliger B Schreiber K Delp K Meissner M Hammers S Reichert T. Downregulation of the constitutive tapasin expression in human tumor cells of distinct origin and its transcriptional upregulation by cytokines. Tissue Antigens (2001) 57(1):39–45. doi: 10.1034/j.1399-0039.2001.057001039.x 11169257

[23] Bennett EM Bennink JR Yewdell JW Brodsky FM. Cutting edge: Adenovirus E19 has two mechanisms for affecting class I MHC expression. J Immunol (1999) 162(9):5049–52. 10227971

[24] Li L Muzahim Y Bouvier M. Crystal structure of adenovirus E3-19K bound to HLA-A2 reveals mechanism for immunomodulation. Nat Struct Mol Biol (2012) 19(11):1176–81. doi: 10.1038/nsmb.2396 PMC3492506 23042604

[25] Harvey IB Wang X Fremont DH. Molluscum contagiosum virus MC80 sabotages MHC-I antigen presentation by targeting tapasin for ER-associated degradation. PloS Pathog (2019) 15(4):e1007711. doi: 10.1371/journal.ppat.1007711 31034515 PMC6508746

[26] Hasim A Abudula M Aimiduo R Ma JQ Jiao Z Akula G. Post-transcriptional and epigenetic regulation of antigen processing machinery (APM) components and HLA-I in cervical cancers from uighur women. PloS One (2012) 7(9):e44952. doi: 10.1371/journal.pone.0044952 23024775 PMC3443204

[27] Thuring C Geironson L Paulsson K. Tapasin and human leukocyte antigen class I dysregulation correlates with survival in glioblastoma multiforme. Anticancer Agents Med Chem (2014) 14(8):1101–9. doi: 10.2174/1871520614666140825110402 25175688

[28] Thuring C Follin E Geironson L Freyhult E Junghans V Harndahl M. HLA class I is most tightly linked to levels of tapasin compared with other antigen-processing proteins in glioblastoma. Br J Cancer (2015) 113(6):952–62. doi: 10.1038/bjc.2015.297 PMC4578088 26313662

[29] Shionoya Y Kanaseki T Miyamoto S Tokita S Hongo A Kikuchi Y. Loss of tapasin in human lung and colon cancer cells and escape from tumor-associated antigen-specific CTL recognition. Oncoimmunology (2017) 6(2):e1274476. doi: 10.1080/2162402X.2016.1274476 28344889 PMC5353923

[30] Jiang Q Pan HY Ye DX Zhang P Zhong LP Zhang ZY. Downregulation of tapasin expression in primary human oral squamous cell carcinoma: association with clinical outcome. Tumour Biol (2010) 31(5):451–9. doi: 10.1007/s13277-010-0054-4 20532727

[31] Dissemond J Kothen T Mors J Weimann TK Lindeke A Goos M. Downregulation of tapasin expression in progressive human malignant melanoma. Arch Dermatol Res (2003) 295(2):43–9. doi: 10.1007/s00403-003-0393-8 12682852

[32] Sokol L Koelzer VH Rau TT Karamitopoulou E Zlobec I Lugli A. Loss of tapasin correlates with diminished CD8(+) T-cell immunity and prognosis in colorectal cancer. J Transl Med (2015) 13:279. doi: 10.1186/s12967-015-0647-1 26310568 PMC4551690

[33] Muller M Agaimy A Zenk J Ettl T Iro H Hartmann A. The prognostic impact of human leukocyte antigen (HLA) class I antigen abnormalities in salivary gland cancer. A clinicopathological study of 288 cases. Histopathology (2013) 62(6):847–59. doi: 10.1111/his.12086 23611358

[34] Boulanger DSM Eccleston RC Phillips A Coveney PV Elliott T Dalchau N. A mechanistic model for predicting cell surface presentation of competing peptides by MHC class I molecules. Front Immunol (2018) 9:1538. doi: 10.3389/fimmu.2018.01538 30026743 PMC6041393

[35] McShan AC Natarajan K Kumirov VK Flores-Solis D Jiang J Badstubner M. Peptide exchange on MHC-I by TAPBPR is driven by a negative allostery release cycle. Nat Chem Biol (2018) 14(8):811–20. doi: 10.1038/s41589-018-0096-2 PMC6202177 29988068

[36] McShan AC Devlin CA Overall SA Park J Toor JS Moschidi D. Molecular determinants of chaperone interactions on MHC-I for folding and antigen repertoire selection. Proc Natl Acad Sci U.S.A. (2019) 116(51):25602–13. doi: 10.1073/pnas.1915562116 PMC6926029 31796585

[37] Lev A Takeda K Zanker D Maynard JC Dimberu P Waffarn E. The exception that reinforces the rule: crosspriming by cytosolic peptides that escape degradation. Immunity (2008) 28(6):787–98. doi: 10.1016/j.immuni.2008.04.015 PMC2587262 18549799

[38] Lemke H Hammerling GJ Hammerling U. Fine specificity analysis with monoclonal antibodies of antigens controlled by the major histocompatibility complex and by the Qa/TL region in mice. Immunol Rev (1979) 47:175–206. doi: 10.1111/j.1600-065X.1979.tb00293.x 398326

[39] Hammerling GJ Rusch E Tada N Kimura S Hammerling U. Localization of allodeterminants on h-2Kb antigens determined with monoclonal antibodies and h-2 mutant mice. Proc Natl Acad Sci U.S.A. (1982) 79(15):4737–41. doi: 10.1073/pnas.79.15.4737 PMC346752 6181513

[40] Markwardt ML Kremers GJ Kraft CA Ray K Cranfill PJ Wilson KA. An improved cerulean fluorescent protein with enhanced brightness and reduced reversible photoswitching. PloS One (2011) 6(3):e17896. doi: 10.1371/journal.pone.0017896 21479270 PMC3066204

[41] Schwanhausser B Busse D Li N Dittmar G Schuchhardt J Wolf J. Global quantification of mammalian gene expression control. Nature (2011) 473(7347):337–42. doi: 10.1038/nature10098 21593866

[42] Wisniewski JR Hein MY Cox J Mann M. A "proteomic ruler" for protein copy number and concentration estimation without spike-in standards. Mol Cell Proteomics (2014) 13(12):3497–506. doi: 10.1074/mcp.M113.037309 PMC4256500 25225357

[43] Probst HC Tschannen K Gallimore A Martinic M Basler M Dumrese T. Immunodominance of an antiviral cytotoxic T cell response is shaped by the kinetics of viral protein expression. J Immunol (2003) 171(10):5415–22. doi: 10.4049/jimmunol.171.10.5415 14607945

[44] Thomas C Tampe R. MHC I assembly and peptide editing - chaperones, clients, and molecular plasticity in immunity. Curr Opin Immunol (2021) 70:48–56. doi: 10.1016/j.coi.2021.02.004 33689959

[45] Williams AP Peh CA Purcell AW McCluskey J Elliott T. Optimization of the MHC class I peptide cargo is dependent on tapasin. Immunity (2002) 16(4):509–20. doi: 10.1016/S1074-7613(02)00304-7 11970875

[46] Garbi N Tan P Diehl AD Chambers BJ Ljunggren HG Momburg F. Impaired immune responses and altered peptide repertoire in tapasin-deficient mice. Nat Immunol (2000) 1(3):234–8. doi: 10.1038/79775 10973281

[47] Grandea AG 3rd Golovina TN Hamilton SE Sriram V Spies T Brutkiewicz RR. Impaired assembly yet normal trafficking of MHC class I molecules in tapasin mutant mice. Immunity (2000) 13(2):213–22. doi: 10.1016/S1074-7613(00)00021-2 10981964

[48] Purcell AW Gorman JJ Garcia-Peydro M Paradela A Burrows SR Talbo GH. Quantitative and qualitative influences of tapasin on the class I peptide repertoire. J Immunol (2001) 166(2):1016–27. doi: 10.4049/jimmunol.166.2.1016 11145681

[49] Turnquist HR Kohlgraf KG McIlhaney MM Mosley RL Hollingsworth MA Solheim JC. Tapasin decreases immune responsiveness to a model tumor antigen. J Clin Immunol (2004) 24(4):462–70. doi: 10.1023/B:JOCI.0000029118.51587.d9 15163903

[50] Racanelli V Leone P Frassanito MA Brunetti C Perosa F Ferrone S. Alterations in the antigen processing-presenting machinery of transformed plasma cells are associated with reduced recognition by CD8+ T cells and characterize the progression of MGUS to multiple myeloma. Blood (2010) 115(6):1185–93. doi: 10.1182/blood-2009-06-228676 PMC2826230 20008301

[51] Carretero FJ Del Campo AB Flores-Martin JF Mendez R Garcia-Lopez C Cozar JM. Frequent HLA class I alterations in human prostate cancer: molecular mechanisms and clinical relevance. Cancer Immunol Immunother (2016) 65(1):47–59. doi: 10.1007/s00262-015-1774-5 26611618 PMC11029306

[52] Romero I Garrido C Algarra I Chamorro V Collado A Garrido F. MHC intratumoral heterogeneity may predict cancer progression and response to immunotherapy. Front Immunol (2018) 9:102. doi: 10.3389/fimmu.2018.00102 29434605 PMC5796886

[53] Goncalves G Mullan KA Duscharla D Ayala R Croft NP Faridi P. IFNgamma modulates the immunopeptidome of triple negative breast cancer cells by enhancing and diversifying antigen processing and presentation. Front Immunol (2021) 12:645770. doi: 10.3389/fimmu.2021.645770 33968037 PMC8100505

