# Supplementary material for: Tapasin-mediated editing of the MHC I immunopeptidome is epitope specific and dependent on peptide off-rate, abundance, and level of tapasin expression
Source: Front Immunol. 2022 Oct 31;13:956603. doi: 10.3389/fimmu.2022.956603 (PMC9659924; doi:10.3389/fimmu.2022.956603)
Supplement: Supplementary file 1 [file DataSheet_1.pdf]

Supplementary Figure 1

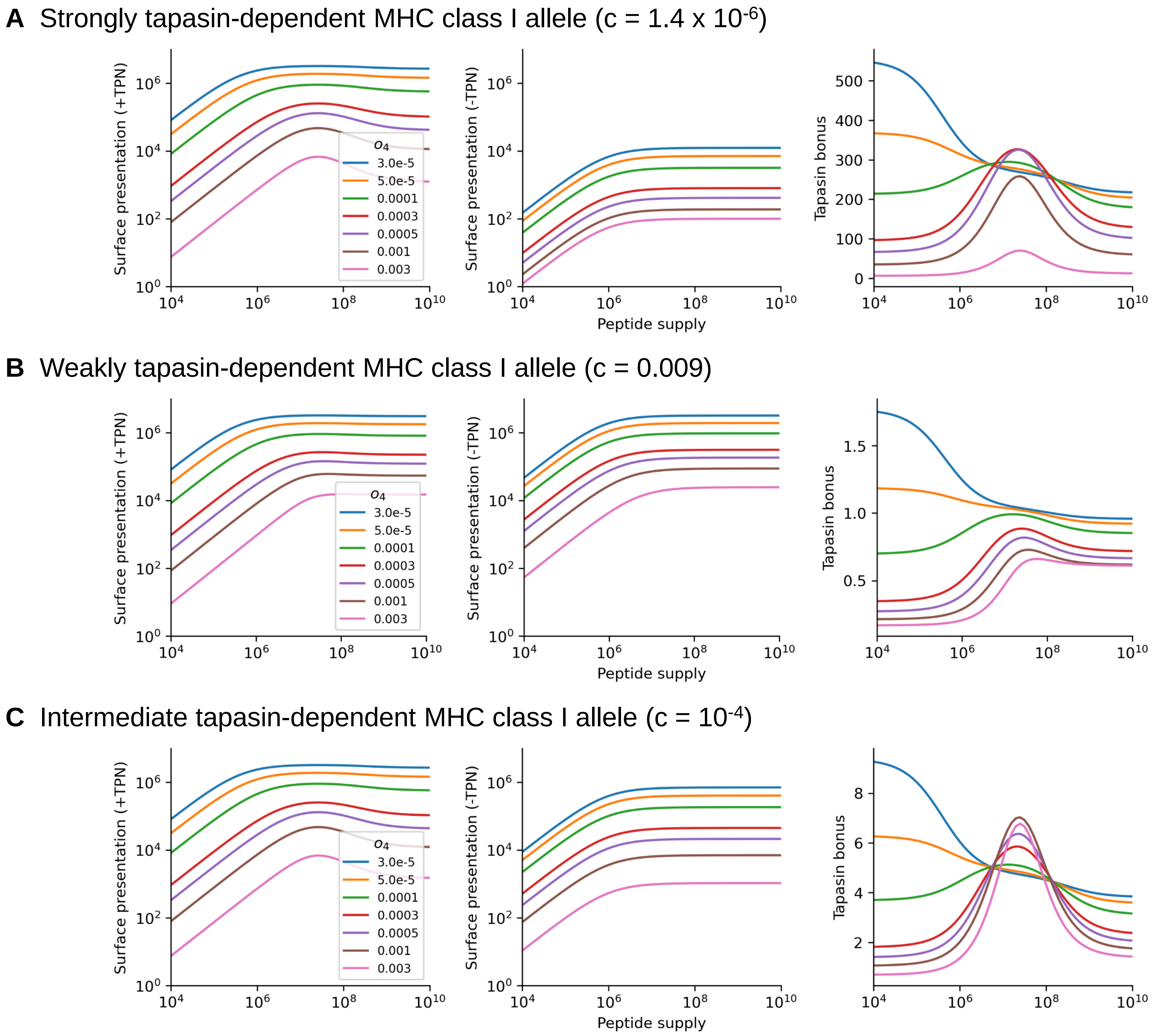

Supplementary Figure 1. Tapasin optimization depends on peptide supply in model simulations of MHC class I.
The *inverse flytrap* model described in Bailey et al. (2016) was simulated with 4 peptides. The first 3 are representative background ("self") peptides of low (o_1), medium (o_2) and high (o_3) affinity/stability for MHC class I, as in the original study. The fourth peptide with various off-rate o_4 represents the endogenous expression of fluorescent protein fusion peptides. Variations in intracellular peptide abundance resulting from cellular and molecular biology factors (e.g. transfection efficiency or ubiquitination) are then explored by varying the supply of the 4th peptide. Equilibrium surface presentation of peptide-MHC complexes is shown for three simulated MHC class I alleles with varying tapasin-dependency: (A) strong (closing rate, c = 1.4 x 10^{-6}; HLA-B*44:02), (B) weak (c = 0.009; HLA-B*44:05) or (C) intermediate (c = 10^{-4}). The equilibrium is calculated by simulating the full dynamical model for 3600000 seconds, so that transients have become negligible. The left-hand columns show "wild-type" (tapasin-competent) presentation, the middle column shows tapasin-negative cells, and the rightmost column shows the simulated tapasin bonus (ratio of wild-type to tapasin-negative presentation).

Supplementary Figure 2

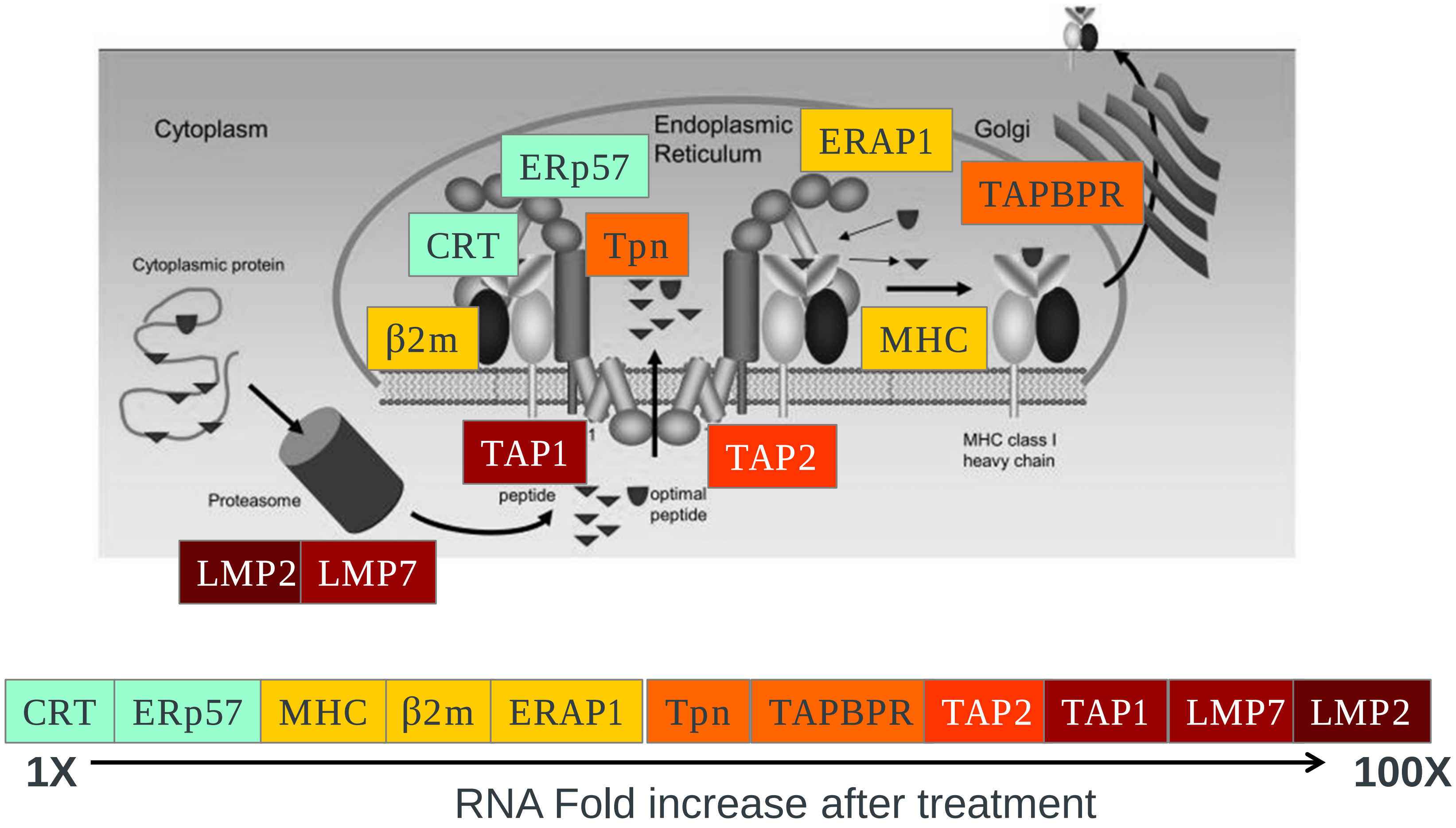

Supplementary Figure 2. Measure of the level of expression of PLC proteins after IFNγ treatment by RT-qPCR.
To determine the increase in MHC I, tapasin and other components of the PLC after IFNγ treatment, fibroblasts were seeded and treated with IFNγ in the same conditions as in the pMHC presentation assay. Cells were harvested one day later by trypsinisation. RNA was extracted immediately from 4 x 10^6 cells using the RNeasy mini kit (Qiagen) including DNase I (Qiagen) treatment. RNA concentration was determined by nanodrop and purity was assessed by nanodrop and Agilent RNA 6000 Nano kit. For reverse transcription, 1 ug of total RNA was transcribed into cDNA using Olido(dT)15 primers (Promega) and GoScriptTM Reverse transcriptase (Promega). Real time polymerase chain reaction was performed using the LightCycler® 480 Real Time PCR System. TaqMan™ Gene Expression Assays consisting of two pre-defined unlabelled PCR primers and a FAM™ or VIC dye-labelled TaqMan™ MGB/NFQ probe (non-fluorescent quencher), all combined into a 20X formulation, were purchased from Applied Biosystems.

Supplementary Figure 3

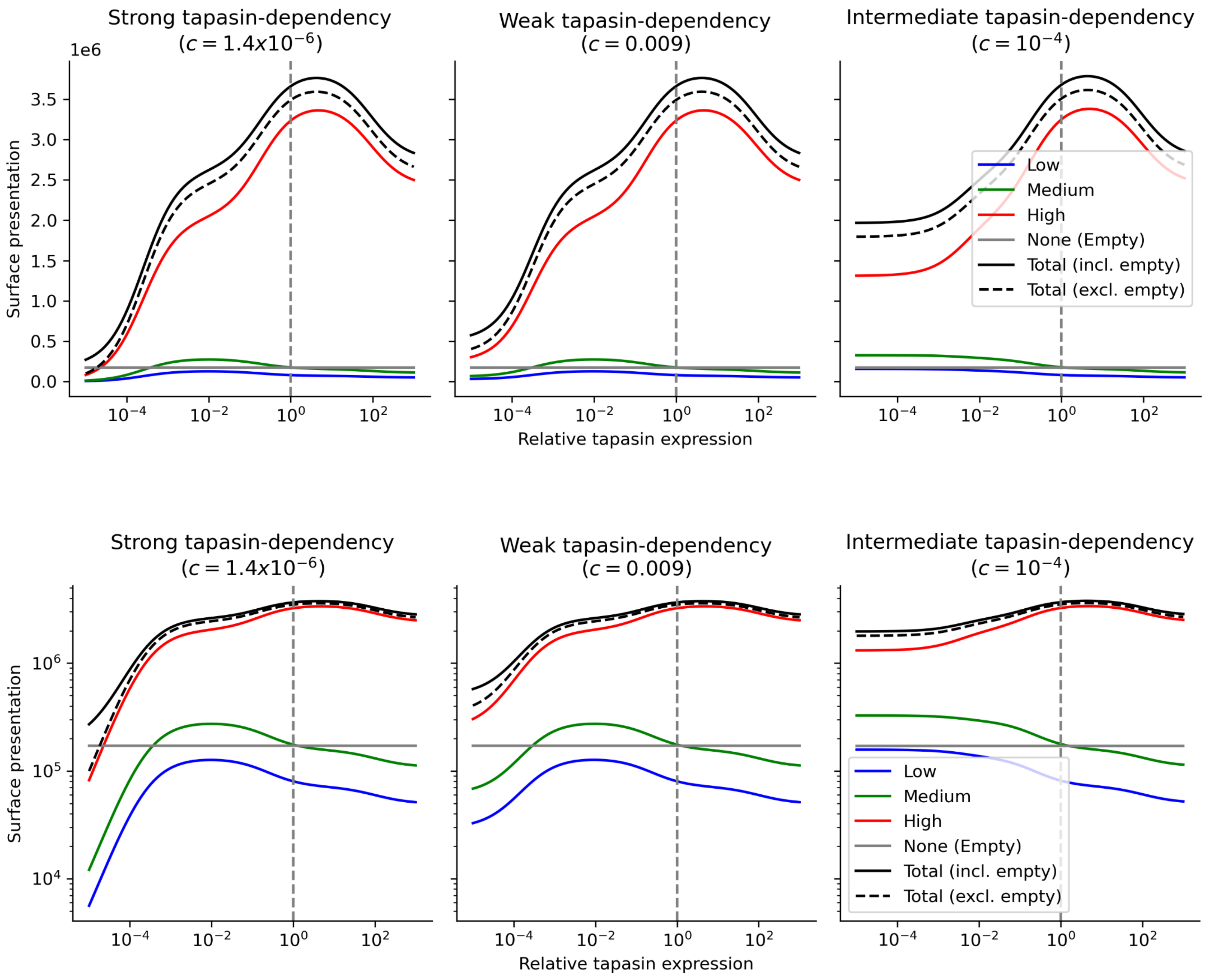

Supplementary Figure 3. Surface presentation is optimized by intermediate tapasin expression in model simulations of MHC class I. The *inverse flytrap* model described in Bailey et al. (2016) was simulated with 3 representative background ("self") peptides of low (o_1), medium (o_2) and high (o_3) affinity/stability for MHC class I, as in the original study. Variations in intracellular tapasin expression are then explored by varying the rate of tapasin supply in the model over a large range. Plotted are surface presentation of low (blue lines), medium (green lines) and high (red lines) affinity peptides, empty MHC molecules (peptide dissociated at the cell surface; grey), and total pMHC complexes (black lines). Equilibrium surface presentation of peptide-MHC complexes is shown for three simulated MHC class I alleles with varying tapasin-dependency, as indicated. The tapasin expression rate in the original model is indicated by the vertical grey dashed line. The equilibrium is calculated by simulating the full dynamical model for 3600000 seconds, so that transients have become negligible. The top and bottom rows of plots show the same data, but plotted on linear and logarithmic scales respectively.

Supplementary Figure 4

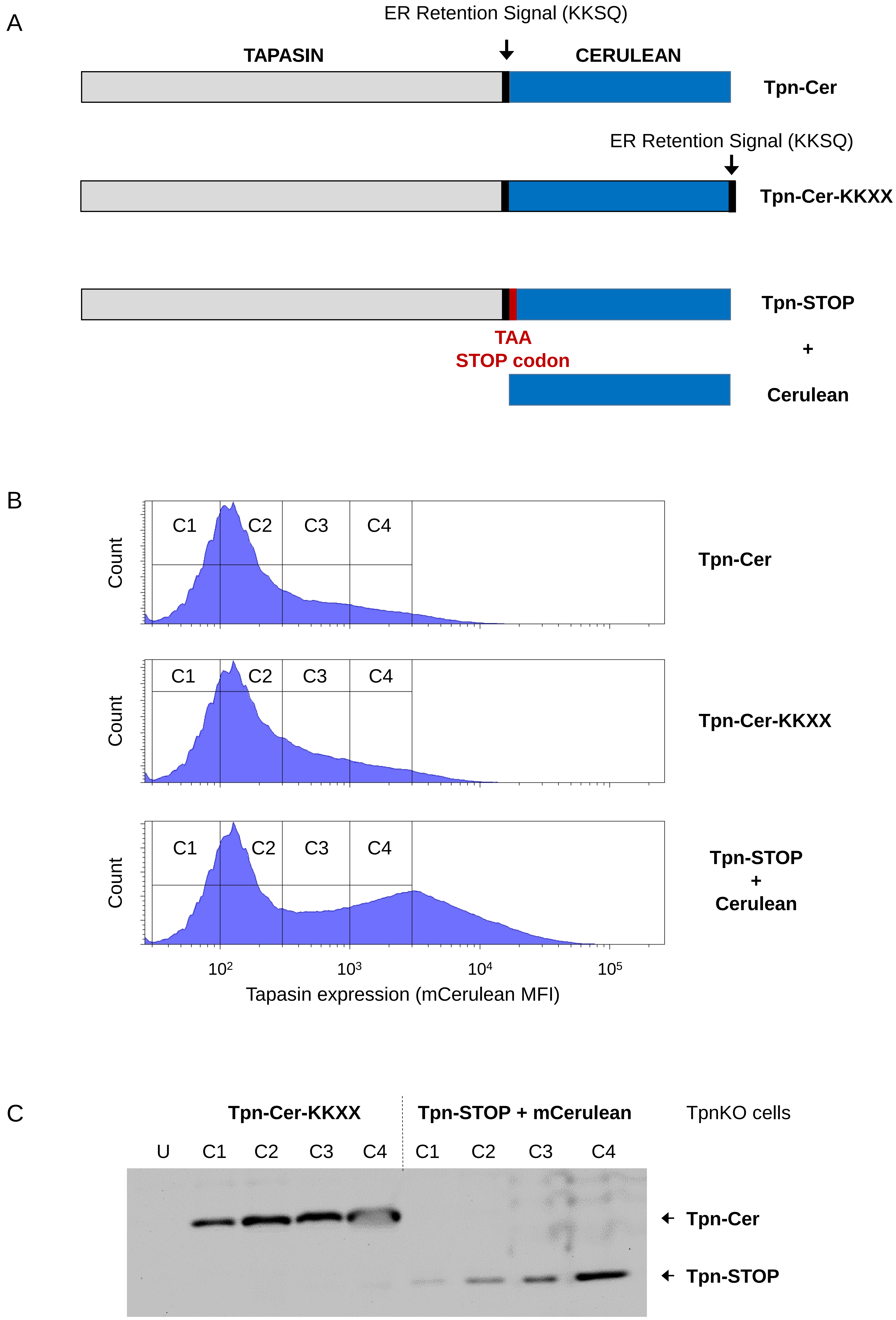

D

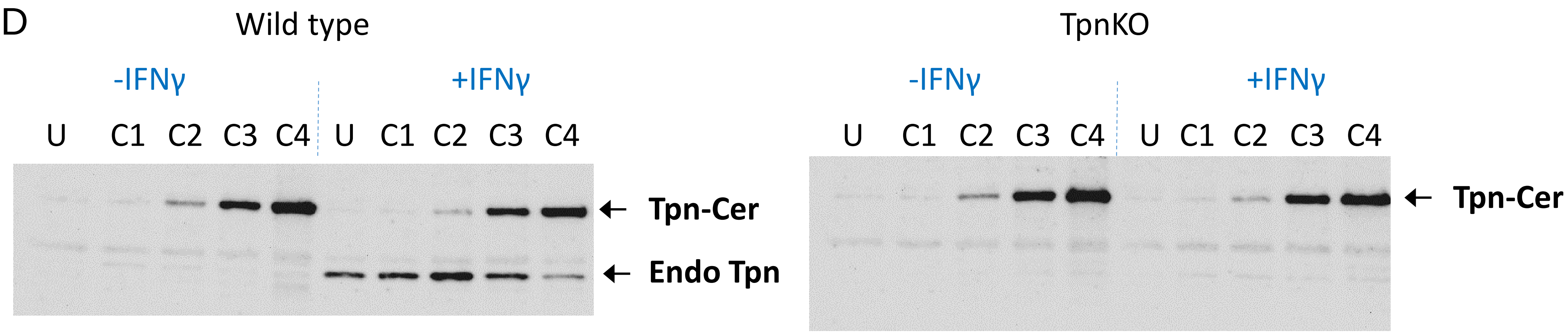

E

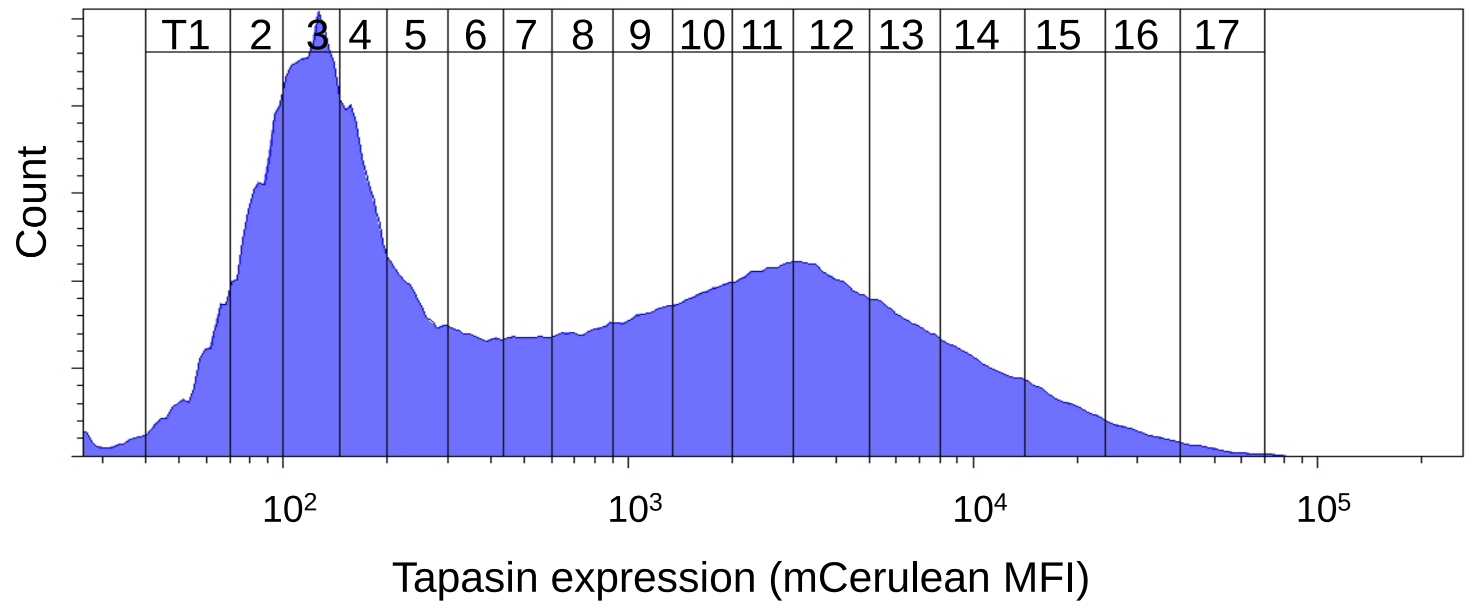

F

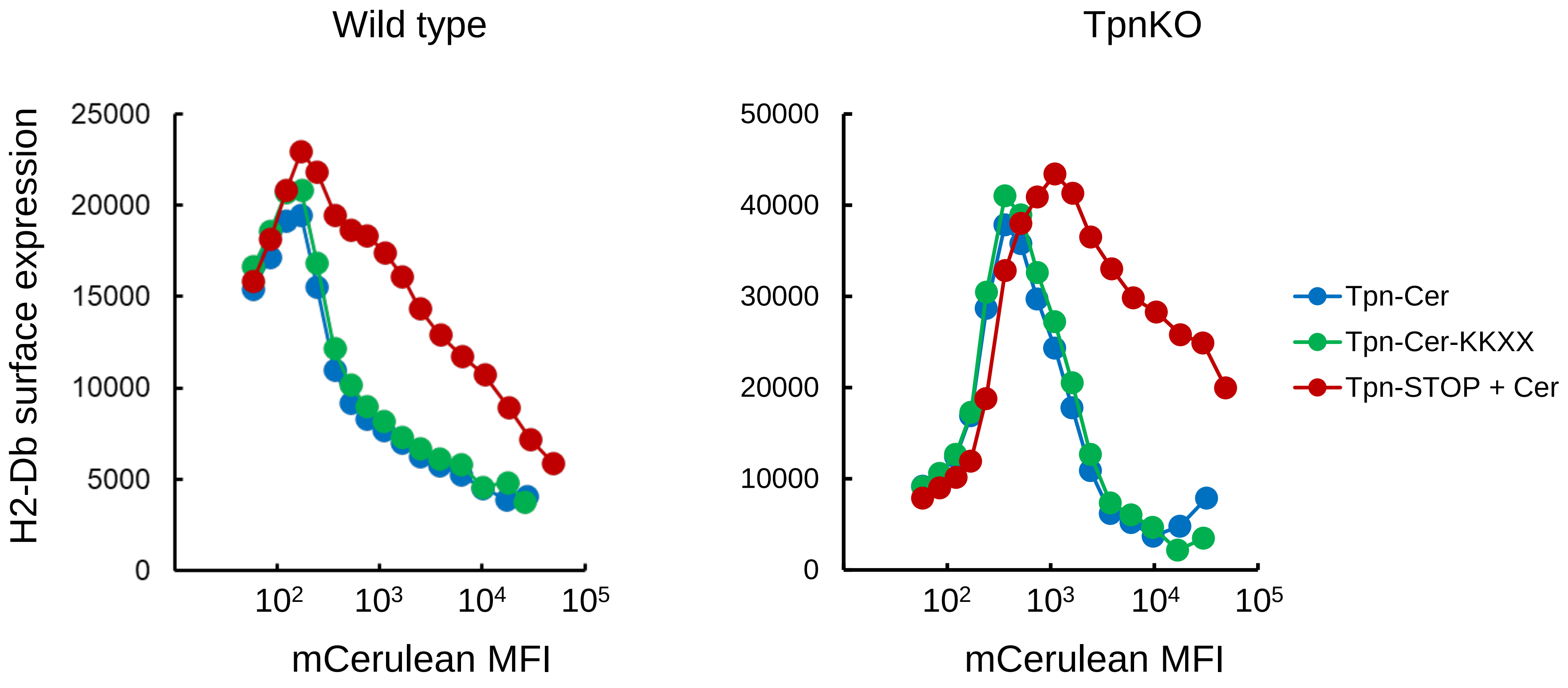

Supplementary Figure 4. (A) Schematic of tapasin constructs. Cerulean was fused to the C-terminus of tapasin as a reporter for the level of expression of the fusion protein measurable via fluorescence intensity. Cerulean was fused at the C-terminus of tapasin to not interfere with the PLC formation, however as tapasin contains an ER retention signal (KKSQ) at its C-terminus, the fusion protein might not be retained in the ER and might have a reduced function in peptide editing, comparable to soluble tapasin. To maintain tapasin location in the ER, the Tpn ER retention signal (KKSQ) was added to the C-terminus of Cerulean (Tpn-Cer-KKXX) in a second construct. As a control, a STOP codon was inserted downstream the ER retention signal and the empty plasmid expressing Cerulean was co-transfected to report indirectly the level of expression of tapasin. (B) The fluorescence intensity of cells transfected with Tpn-STOP + Cerulean was much higher than of Tpn-Cer or Tpn-Cer-KKXX, suggesting that either Tpn-Cer had a shorter half-life than Cerulean or that, due to their proximity in the ER, the fusion proteins were dimerising resulting in quenching of Cerulean. (C) The level of expression of tapasin in TpnKO cells transfected with Tpn-Cer-KKXX (left) or Tpn-STOP + mCer (right) was compared by Western blot analysis. Transfected cells were sorted in 4 gates with increasing Cerulean fluorescence intensity (C1 to C4) as indicated in figure B. U indicates untransfected TpnKO lysates. Lysates from 50,000 sorted cells were run on SDS-PAGE; separated proteins were transferred on nitrocellulose membrane and tapasin was identified by using the anti-tapasin 5D3 antibody (Biolegend) and a goat-anti-mouse-HRP conjugate (Invitrogen). Tpn-STOP (same as endogenous tapasin) appeared as a 48kDa protein while Tpn-Cer appeared as a 70kDa protein. Tapasin expression was clearly higher in cells transfected with Tpn-Cer-KKXX than in the co-transfected samples, suggesting that Cerulean was quenched in the ER compartment and did not truly reflect the amount of tapasin. (D) The expression of transfected tapasin was similar in wild type (left) and TpnKO fibroblasts (right), treated or not with IFNγ, as it is under the control of the CMV promoter, which is not regulated by IFNγ. Cells were transfected with the Tpn-Cer construct, as Tpn-STOP overlaps with endogenous tapasin and would not have allowed direct comparison. (E) Cells transfected with Tpn-STOP and Cerulean were gated in a series of gates of increasing mCerulean MFI levels (increasing tapasin expression), T1 to T17. (F) Wild type (left panel) and TpnKO (right panel) fibroblasts were transfected with Tpn-Cer, Tpn-Cer-KKXX or Tpn-STOP + Cerulean. Cells were stained with the B22 antibody to measured total H2-Db surface expression, which was plotted at increasing levels of tapasin expression reported by the Cerulean MFI in gates T1 to T17. MHC I expression first increased as tapasin expression increased, reached a maximum and then declined in wild type and in TpnKO cells. While Tpn-Cer and Tpn-Cer-KKXX behaved in a very similar way, the decline seen in Tpn-STOP + Cer transfected cells was delayed, which is consistent with the observation that tapasin expression was lower for similar Cerulean fluorescence intensity.

Supplementary Figure 5

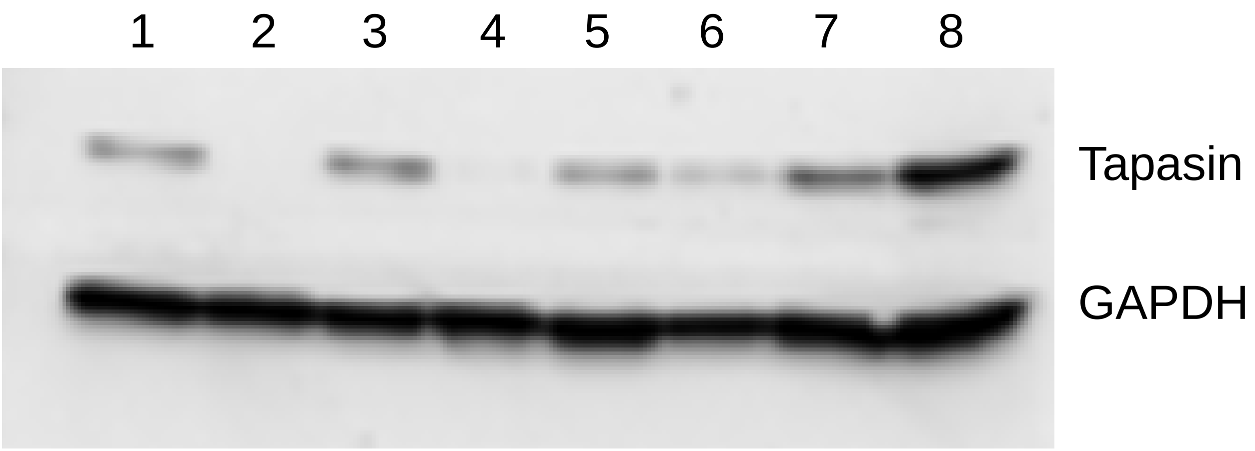

Supplementary Figure 5. Semi-quantitation of tapasin expressed in a selection of human cancer cell lines. (1) HeLa, (2) .220, (3) KG1, (4) HL60, (5) LUDLU-1, (6) 5637, (7) COR-L105 and (8) Sk-LU-1. Lysates from 50,000 cells were loaded on a 12.5% SDS-PAGE gel. Separated proteins were transferred on a nitrocellulose membrane. Tapasin was revealed using the TPN-B9 anti-human tapasin antibody from Santa Cruz and an anti-GAPDH antibody from Abcam was used as a loading control. Different levels of tapasin downregulation are observed as well as overexpression in the Sk-LU-1 lung adenocarcinoma cell line.

Supplementary Figure 6

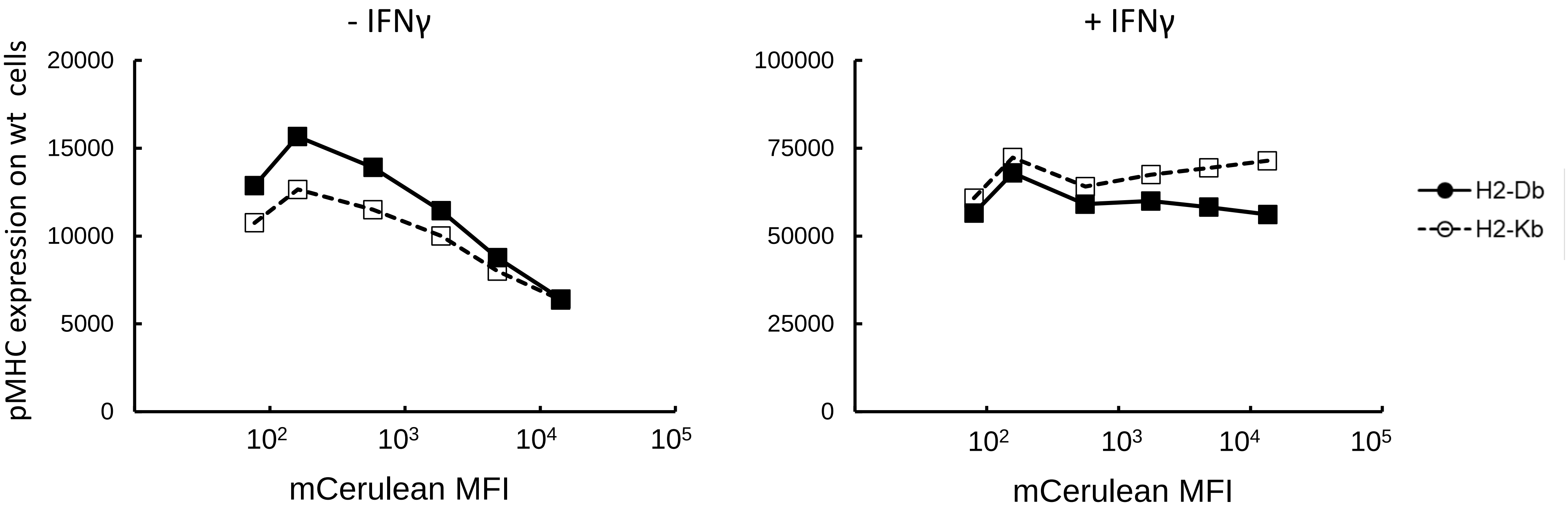

Supplementary Figure 6. pMHC surface presentation on wild type fibroblasts at increasing levels of tapasin expression. Cells were co-transfected with plasmids expressing tapasin and mCerulean reporting indirectly the level of tapasin expression, and divided in 6 gates of increasing expression level (S1 to S6, see Figure 3A). H-2Db (filled circles) and H-2Kb (empty circles dotted line) surface levels on cells expressing increasing amount of transfected tapasin reported by cerulean MFI on the x-axis (untreated cells on the left; IFNγ treated cells on the right).
